# L’épisclérite nodulaire: une manifestation inaugurale inhabituelle de la maladie de Horton

**DOI:** 10.11604/pamj.2015.21.20.6935

**Published:** 2015-05-08

**Authors:** Rim Klii, Wafa Chebbi

**Affiliations:** 1 Service de Médecine Interne et d'Endocrinologie, CHU Fattouma Bourguiba Monastir, 5000 Monastir, Tunisie; 2 Service de Médecine Interne, CHU Taher Sfar Mahdia, 5100 Mahdia, Tunisie

**Keywords:** Episclérite, maladie de Horton, aortite inflammatoire, Episcleritis, Horton disease, inflammatory aortitis

## Image en medicine

Les atteintes oculaires les plus fréquentes au cours de la maladie de Horton sont la névrite optique ischémique antérieure aiguë, l'occlusion des artères rétiniennes et la neuropathie optique ischémique postérieure. L’épisclérite est une manifestation ophtalmologique rare de cette vascularite et exceptionnellement révélatrice. Nous rapportons l'observation d'un patient âgé de 55 ans, adressé pour bilan étiologique d'une épisclérite nodulaire récidivante à bascule. A l'anamnèse, le patient rapportait des céphalées temporales depuis un mois. Il n'y avait pas de notion d'altération de l’état général, ni de sueurs nocturnes, ni de signe de pseudo-polyarthrite rhizomélique, ni d'aphtose buccale et/ou génitale, ni de troubles de transit. L'examen clinique était normal. Le bilan biologique montrait une vitesse de sédimentation à 78 mm à la première heure, une protéine C réactive à 32 mg/l, une fibrinémie à 8,8 g/l et une hyperalpha2-globulinémie. L'enquête infectieuse comportant les sérologies de rickettsioses et virales (virus varicelle-zona, herpès simplex virus, hépatite B et C, virus de l'immunodéficience humaine, Parvovirus B19), ainsi que le Quantiferon était négative. La recherche d'anticorps anti-nucléaires, anti-cytoplasme de polynucléaires neutrophiles, anti-CCP, et du facteur rhumatoïde était négative. La radiographie thoracique était sans anomalies. La tomodensitométrie thoraco-abdominale objectivait un épaississement régulier circonférentiel hypodense de la paroi aortique touchant l'aorte thoracique ascendante et descendante, les troncs supra aortiques, et de l'aorte abdominale. La biopsie d'artère temporale montrait un aspect d'artérite temporale à cellules géantes. Une corticothérapie à la dose de 1 mg/kg/j était instaurée, entrainant une régression de l’épisclérite et des céphalées avec absence de récidive après un recul de 6 mois.

**Figure 1:**
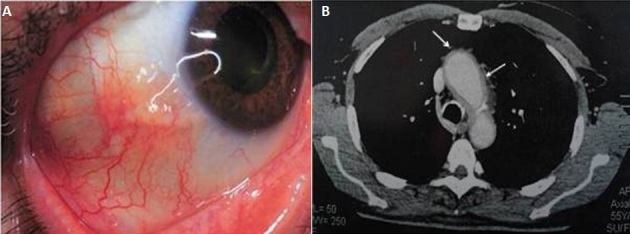
(A) épisclérite nodulaire de l’œil droit; (B) TDM Thoracique en coupe axiale: épaississement régulier circonférentiel hypodense de la paroi aortique touchant l'aorte thoracique ascendante et descendante

